# Impact of Covid-19 on the management of patients with metastatic melanoma

**DOI:** 10.18632/oncotarget.28333

**Published:** 2022-12-29

**Authors:** Michèle Welti, Phil F. Cheng, Joanna Mangana, Mitchell P. Levesque, Reinhard Dummer, Laurence Imhof

**Affiliations:** ^1^Department of Dermatology, University Hospital Zurich (USZ), Zurich 8091, Switzerland; ^2^Faculty of Medicine, University of Zurich (UZH), Zurich 8032, Switzerland; ^3^Institute for Biomedical Engineering, ETH Zurich, Zurich 8092, Switzerland

**Keywords:** metastatic melanoma, Covid-19, Sars-CoV-2, immunotherapy, targeted therapy

## Abstract

The Covid-19 pandemic created new uncertainties in the management of metastatic melanoma patients. In particular, the impact of immunotherapy, targeted therapy, or chemotherapy on the risk of Sars-CoV-2 infection and severity was debated. In this study, we analyzed all patients with metastatic melanoma receiving therapy who developed Covid-19 between February 2020 and February 2022. We retrospectively collected demographic data, cancer-specific parameters, melanoma treatment regimen, comorbidities and Covid-19-specific parameters in these patients. Of the 350 patients with metastatic melanoma, 25 had Covid-19. The median age at the time of Covid-19 diagnosis was 66 years (range 36–86), 10 patients were female, and 15 patients were male. The treatment regimen during infection was immunotherapy in 12 cases, followed by targeted therapy (*n* = 8), chemotherapy (*n* = 2), and TVEC injections, follow-up and palliative therapy in 1 case each. The severity was mild in 17 patients and 8 had a moderate to critical course. Patients with a severe Covid-19 course were often older and had more comorbidities than patients with a mild infection. Many of the patients had a mild Covid-19 course despite having metastatic melanoma and systemic therapy. We therefore recommend continuing systemic therapy whenever possible, even in such exceptional situations as the Covid-19 pandemic.

## INTRODUCTION

For more than two years there was a global pandemic due to the severe acute respiratory syndrome coronavirus 2 (Sars-CoV-2) and the resulting disease, Corona Virus Disease 2019 (Covid-19). Early studies showed that several factors correlated to more severe infection and thus potentially higher mortality. These include the demographic factors of old age, male sex, smoking status and high Eastern Cooperative Oncology Group (ECOG) performance status [1]. Comorbidities with a higher risk for severe disease are obesity, hypertension or other cardiovascular risk factors, diabetes mellitus, respiratory diseases such as chronic obstructive pulmonary disease (COPD) and cancer [2, 3]. Cancer patients belong to the high-risk population in the pandemic due to their immunocompromised status induced by both the cancer itself and the effects of the anti-cancer therapy [3]. Especially chemotherapy with myelosuppressive side-effects is considered a risk factor for the development of severe infection. Therefore, studies from early in the pandemic recommend postponing chemotherapy, while later studies show that chemotherapy did not lead to increased mortality [4, 5]. In contrast, immunotherapy with Immune Checkpoint Inhibitors (ICI) such as Programmed Cell Death Protein 1 (PD-1), Programmed Cell Death Ligand 1 (PD-L1) or Cytotoxic T-Lymphocyte-Associated Protein 4 (CTLA-4) inhibitors were even hypothesized to have a positive effect on Covid-19 and may have a protective factor against severe Sars-CoV-2 infection. This is due to ICI leading to an enhanced immune response through T-cell activation [6–8]. This might be beneficial during Covid-19, as studies showed that severe Sars-CoV-2 infection was associated with lymphocytopenia and CD8+ T-cell depletion [9]. However, the inflammatory response can also lead to increased release of inflammatory cytokines and elevated levels of pre-existing autoantibodies, which affect almost every organ and cause immune related adverse events (irAEs). These can mimic Sars-CoV-2 infection with nonspecific symptoms such as fever, cough, fatigue, or dyspnea. In addition, immune-related pneumonitis presents both clinically and radiologically similar to Covid-19 pneumonia. Thus, clinical differentiation of the two etiologies can be very difficult [6, 9]. There are still many unanswered questions in the management of cancer patients due to the ongoing pandemic.

For melanoma patients, studies showed a significant delay in the diagnosis of new melanomas since the start of the pandemic. In order to minimize the burden on the healthcare system and physical contact, many medical visits, such as skin cancer screenings were postponed. For this reason, newly detected tumors had significantly higher tumor thickness and mitotic rates and overall higher TNM stage at diagnosis [6, 10–13]. However, the pandemic also had implications for the treatment of patients with metastatic melanoma (AJCC 8th Edition Stage 3 and 4). These patients are usually treated with targeted therapy (TT) or immunotherapy (IT), in addition to surgery or radiotherapy when appropriate [14]. As mentioned above, the uncertainties of treatment with ICI during the Covid-19 pandemic also affected patients with metastatic melanoma. There is ongoing research on the optimal management of this patient population during the pandemic. Most of the authors recommend continuing immunotherapy without interruption if possible. They argue that almost no patients have developed Covid-19, and there is increased risk for tumor progression in patients discontinuing their melanoma therapy [9, 10, 15]. However, those studies analyzed all melanoma patients undergoing therapy, including those without Sars-CoV-2 infection, in a very short time period of one to two months. They focused on the infection rate, but since a maximum of nine of these patients actually had Covid-19, no conclusions about the severity and impact of Covid-19 on melanoma therapy could actually be made.

In our study, we aimed to analyze our experience with patients undergoing treatment for metastatic melanoma while testing positive for Sars-CoV-2. A special focus was given to the severity of Covid-19 in context to various comorbidities and therapies.

## RESULTS

### Patient characteristics

114 patients with stage 3 melanoma and 236 patients with stage 4 melanoma were treated between February 2020 and February 2022. Of these 350 total patients, 25 (7.1%) patients were found to have Sars-CoV-2 infection. Ten patients were female and 15 patients were male. At the time of Covid-19 detection, the median age was 66 years (range, 36–86).

The treatment regime at the time of the Sars-CoV-2 infection was immune-checkpoint-inhibition (anti-PD-(L)1 monotherapy or combination therapy) in 12 patients, targeted therapy (BRAF- and MEK-Inhibitors) in 8 patients, chemotherapy in 2 patients and talimogene laherparepvec (TVEC) injections in 1 patient. Two patients received therapy within the scheduled time but were undergoing follow up or palliative care at the time of Covid-19 diagnosis. At the last follow-up in March 2022, 19 patients were still alive, 5 patients died of melanoma, and 1 patient died of Covid-19. Patient characteristics regarding melanoma and melanoma therapy during Covid-19 are shown in Table 1.

**Table 1:** Patient characteristics regarding melanoma and melanoma therapy during Covid-19

Patient characteristics		*n* = 25^1^
Age (years)	Diagnosis Melanoma	55 (24–77)^2^
	Diagnosis Covid-19	66 (36–86) ^2^
Sex	Female	10 (40%)
	Male	15 (60%)
AJCC 8th Edition Stage	IIIB	5 (20%)
	IIIC	5 (20%)
	IV	15 (60%)
Morphology	Nodular melanoma (NM)	12 (46%)
	Superficial spreading melanoma (SSM)	7 (28%)
	Malignant melanoma NOS (not Otherwise Specified)	4 (16%)
	Acral lentiginous melanoma (ALM)	1 (4.0%)
	Mucosal lentiginous melanoma	1 (4.0%)
	Lentigo maligna melanoma (LMM)	0 (0%)
Breslow Thickness (mm)		2.60 (0.70–15.00)^2^ *2 unknown*
BRAF Mutation Status	V600 mutated	14 (56%)
	Wild	8 (32%)
	Unknown	3 (12%)
Treatment during Covid-19	Immunotherapy	12 (48%)
	Targeted Therapy	8 (32%)
	Chemotherapy	2 (8%)
	TVEC Injections	1 (4%)
	Follow-up	1 (4%)
	Palliative	1 (4%)
Alive/Death	Alive	19 (76%)
	Death from Melanoma	5 (20%)
	Death from Covid-19	1 (4%)

^1^ *n* (%); ^2^Median (Range).

### Severity of Covid-19

In our patient cohort, 17 (68%) of the 25 patients had a mild course of Covid-19. Six (24%) patients had a moderate course, one (4%) had a severe course and one (4%) had a critical course of disease. At the time of the infection, 18 (72%) patients were unvaccinated.

**Figure 1:**
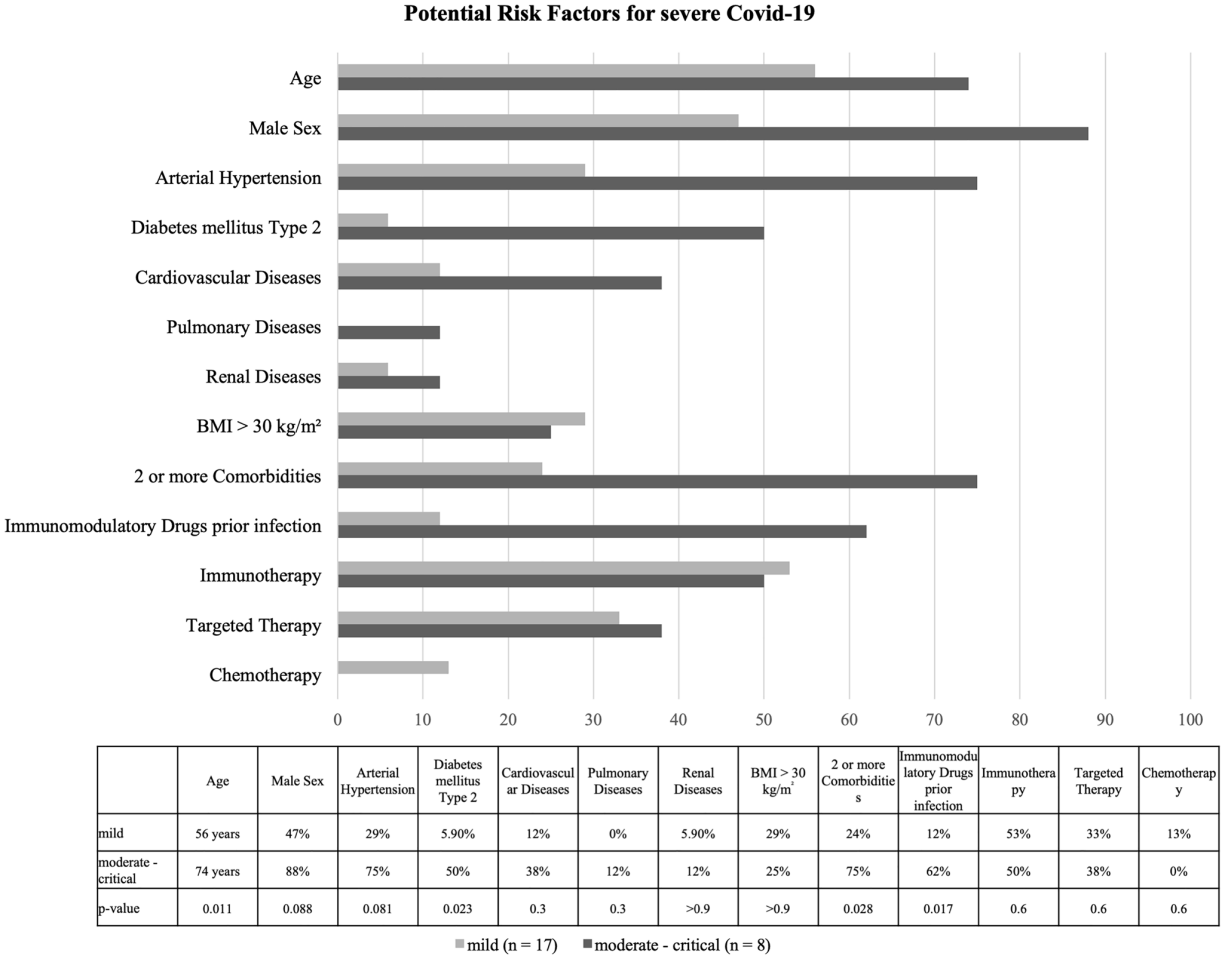
Demographic factors, comorbidities and therapy regime of the patients in percentage, age in years. The difference between the two groups is statistically significant (*p* < 0.05) for age, diabetes mellitus, more than two comorbidities and use of immunomodulatory drugs prior to infection. Data calculated using Fisher’s exact test, Wilcoxon rank sum test, Wilcoxon rank sum exact test.

Factors associated with a moderate to critical infection included advanced age (*p* = 0.011), type 2 diabetes mellitus (*p* = 0.023), the presence of two or more comorbidities (*p* = 0.028) and the use of immunomodulatory drugs (*p* = 0.017). The median age in the mild group was 56 years (range, 36–86) versus 74 years (range, 54–80) in the patients with a moderate – critical course. Besides the significant differences, we could see a trend (*p* = 0.088) towards a gender difference. Of 10 women, 9 (90%) had a mild course, whereas among men only half had a mild course (*n* = 8, 53%). Seven (88%) of the eight patients with a moderate – critical infection were male. This tendency is also visible in arterial hypertension (*p* = 0.081): only five (29%) patients with a mild course had preexisting arterial hypertension, compared with six (75%) in the group of patients with at least a moderate course. We found no association with vaccination status, pulmonary or renal risk factors, laboratory parameters, or type of cancer treatment in our cohort. Figure 1 shows the most common potential risk factors in our cohort.

Six patients (24%) required hospitalization due to Covid-19. Four (16%) of them required standard care and were hospitalized for 4 to 6 days, and 2 (8%) patients required intensive care for a length of 24 and 29 days, respectively. Delay of therapy due to active Sars-CoV-2 infection occurred in 8 (32%) patients. The median delay in melanoma treatment in these patients was 14 days (range, 14–36) and the treatment was discontinued in three patients.

### Immunotherapy

Twelve patients were on immunotherapy while they had Covid-19, 8 (67%) of them with a mild course, 3 (25%) with a moderate course, and 1 (8%) with a severe course. The median age was 48 years (range, 37–86) in the mild group versus 74 years (range, 72–80) in the moderate and severe group. Most of the patients (*n* = 6, 75%) with a mild course had none or one comorbidity; whereas, all patients with a moderate and severe course (*n* = 4, 100%) had two or more comorbidities. The most common comorbidity in all groups was arterial hypertension, followed by type 2 diabetes mellitus, other cardiovascular diseases and obesity. Delay in therapy occurred in 5 (40%) patients, with a median duration of 14 days in the mild group versus 48 days in the moderate and severe group. Progressive disease was registered in one patient with therapy delay at the last follow-up in February 2022; one had a stable disease and three had complete remissions. At the time of this study, 11 of the 12 patients undergoing immunotherapy were alive, and one patient died from melanoma. Details are given in Table 2.

**Table 2:** Overview of patients receiving immunotherapy according to the severity of Covid-19

Immunotherapy, *n* = 12				
		Mild *n* = 8^1^	Moderate – Severe *n* = 4^1^	*p*-value^2^
Vaccination	Yes	2 (40%)	1 (25%)	>0.9
	Unknown	3	0	
Age (years)	Median^3^	48 (37–86)	74 (72–80)	0.049
	Mean^4^	54 (37–86)	75 (72–80)	
Sex	Female	4 (50%)		0.2
	Male	4 (50%)	4 (100%)	
Potential Risk Factors for Covid-19	Arterial Hypertension	3 (38%)	4 (100%)	0.081
	Diabetes mellitus Type 2	1 (12%)	3 (75%)	0.067
	Cardiovascular Comorbidities	1 (12%)	2 (50%)	0.2
	Pulmonary Diseases			
	Renal Diseases		1 (25%)	0.3
	BMI > 30 kg/m^2^	2 (25%)	1 (25%)	>0.9
	ECOG > 2		*unknown*	
	Immunomodulatory Drugs	1 (12%)	2 (50%)	0.2
Number of Comorbidities per Patient	0–1	6 (75%)	0 (0%)	0.061
	2 or more	2 (25%)	4 (100%)	
Need of Hospitalization	Yes		3 (75%)	0.018
	Need of ICU		1 (25%)	
	Duration, days^3^		5 (4–24)	
Therapy Delay	Yes	3 (38%)	2 (50%)	>0.9
	Duration, days^3^	14 (14–17)	48 (36–59)	
Patient Alive	Yes	7 (88%)	4 (100%)	>0.9
	No	1 (12%), died from melanoma		

^1^ *n* (%); ^2^Fisher’s exact test; Wilcoxon rank sum test; ^3^Median (range); ^4^Mean (range).

### Targeted therapy

Eight patients received targeted therapy during their Sars-CoV-2 infection. The infection was mild in five patients, moderate in two patients, and critical in one patient. Two patients were female and both had mild infection. Age was similar in both groups. Most of the patients (*n* = 4, 80%) with a mild course had no or one comorbidity. More comorbidities were found in patients with a moderate to critical infection. The patient with the critical infection for example had three concomitant diseases, namely arterial hypertension, other cardiovascular diseases and obesity. Additionally, the patient used immunomodulatory drugs prior infection. This patient required intensive care for 29 days and died from Covid-19. Melanoma therapy was solely interrupted in this patient with the critical Sars-CoV-2 infection. Details in Table 3.

**Table 3:** Overview of patients receiving targeted therapy according to the severity of Covid-19

Targeted therapy, *n* = 8				
		Mild *n* = 5^1^	Moderate – Critical *n* = 3^1^	*p*-value^2^
Vaccination	Yes	0 (0%)	1 (33%)	0.4
Age (years)	Median^3^	56 (41–69)	54 (54–76)	0.8
	Mean^4^	55 (41–69)	61 (54–76)	
Sex	Female	2 (40%)	0 (0%)	0.5
	Male	3 (60%)	3 (100%)	
Potential Risk Factors for Covid-19	Arterial Hypertension	1 (20%)	1 (33%)	>0.9
	Diabetes mellitus Type 2		1 (33%)	0.4
	Cardiovascular Comorbidities		1 (33%)	0.4
	Pulmonary Diseases		1 (33%)	0.4
	Renal Diseases			
	BMI > 30 kg/m^2^	1 (20%)	1 (33%)	>0.9
	ECOG > 2	unknown	unknown	
	Immunomodulatory Drugs	1 (20%)	3 (100%)	0.14
Number of Comorbidities per Patient	0–1	4 (80%)	1 (33%)	0.5
	2 or more	1 (20%)	2 (67%)	
Need of Hospitalization	Yes	1 (20%)	1 (33%)	>0.9
	Need of ICU		1 (33%)	
	Duration, days^3^	6 (6–6)	29 (29–29)	
Therapy Delay	Yes		1 (33%), therapy stop	0.4
Patient Alive	Yes	3 (60%)	2 (67%)	>0.9
	No	2 (40%), died from melanoma	1 (33%), died from Covid-19	

^1^ *n* (%); ^2^Fisher’s exact test; Wilcoxon rank sum test; ^3^Median (range); ^4^Mean (range).

## DISCUSSION

The pandemic has led to many uncertainties in patient management at all levels. In particular, the management of individuals with cancer, especially those on systemic therapies. In this study, we aimed to analyze our experience with patients suffering from Sars-CoV-2 infection while receiving treatment for metastatic melanoma. Only 7.1% of our patients with metastatic melanoma, or 25 out of 350 patients, had documented Covid-19. This is very low in terms of the rate of disease in the general population. Nationwide, 2’801’923 cases were reported by the end of February 2022, representing 32% of the Swiss population [16]. In our vulnerable patient population, most (68%) had a mild, i.e., asymptomatic or with upper respiratory symptoms, infection. This was despite the fact that 80% of patients were on either direct treatment with immunotherapy (48%) or targeted therapy (32%). At that time, only a few of the patients had been fully vaccinated. Double vaccination was present in only four (16%) patients. This is due to the fact that, on one hand, no vaccination was available at the beginning of the pandemic and, on the other hand, many patients deliberately decided against vaccination.

Twelve patients in our clinic were on immunotherapy at the time of Sars-CoV-2 infection. Most of them (*n* = 8, 67%) experienced a mild course of infection. Only three patients (25%) had a moderate course and one patient (8%) had a severe course. We noticed that the patients with a mild course were significant younger on average than those with a moderate or severe course. They also had fewer comorbidities. Patients with a moderate or severe course had at least two concomitant diseases, mainly arterial hypertension, followed by type 2 diabetes mellitus. The group of Rogiers and colleagues already analyzed the clinical impact of Covid-19 on 110 patients with cancer treated with immune checkpoint inhibition. Most patients had an asymptomatic or mild infection, as in our case. Hospital admission was required for 32% of the patients. Moreover, the few patients who died did not die from Covid-19. Factors associated with hospitalization were combined immunotherapy, ECOG >2, therapy with corticosteroids, dyspnea, and lymphocytopenia. They could not make any correlation with the risk factors cardiovascular disease, older age or male sex in relation to mortality. They concluded that patients with cancer are at a higher risk of severe Covid-19, but that the treatment with ICI does not appear to be an additional risk factor for a severe Covid-19 infection [7]. In contrast, Kuderer and colleagues from the Covid-19 and Cancer Consortium (CCC19) did not come to a clear conclusion if ICI has a negative impact on disease severity [6]. The study by Rabilotti and colleagues even found an association of ICI as a risk factor for severe outcomes, which was independent of age, cancer type and other comorbid conditions [4].

Delay of immunotherapy occurred in five (42%) patients, regardless of severity. This resulted in a prolongation of the therapy cycle of a median of 14 days in the mild group and 48 days in the moderate and severe group. This delay was mainly due to the active infection at the time of the scheduled immunotherapy or the isolation requirements. Pala and colleagues evaluated treatment delay in their patients with advanced melanoma receiving immunotherapy during the pandemic. They observed a median treatment delay of 4 weeks in 29% of patients. The treatment delay was intended to prevent hospital visits in half of the patients because of their higher risk of severe Covid-19 (e.g., because of high age or comorbidities). Treatment was only stopped in patients who had been on therapy for at least four months with a stable disease before delay [15]. The follow-up of our patients showed that the treatment delay in immunotherapy did not have any negative impact on the course of melanoma disease. Of the five patients with therapy delay, four (80%) were either in complete remission or stable disease at last follow-up. With these observations and considering the half-life and steady state of the ICI, such therapeutic delays are justifiable in our view. For example, the half-life of Nivolumab is 25 days with a steady state reached after 12 weeks, and the half-life of Pembrolizumab is 14–27.3 days with a steady state reached after approximately 18 weeks [17].

A delay in therapy was not recorded in our patients with targeted therapy. Due to the oral form of drug administration, there was no interruption of therapy in these patients, which has turned out to be a major advantage in the pandemic. Otherwise, the experience in this therapy group was similar to the experience in the immunotherapy group. Of the eight patients who developed Covid-19 during treatment with targeted therapy, five (63%) had a mild course, two (25%) had a moderate course, and one (12%) patient had a critical course which resulted in death. However, with a very small number of patients, we could not detect any underlying cause for the different severity of Sars-CoV-2 infection in our patients receiving targeted therapy. To date, few studies have analyzed the sole impact of targeted therapy on the severity of Covid-19. However, Yekeduz and colleagues concluded in their univariable meta-analysis that targeted therapy did not increase the risk for severe infection [18]. The European Society for Medical Oncology (ESMO) also did not recommend discontinuation of therapy if possible, because, like immunotherapy, TT has not been linked to Covid-19 associated mortality up to today [19].

Since most patients in both the immunotherapy group and the targeted therapy group had a mild course, we do not expect melanoma therapy to have an impact on the severity of Covid-19. So which factors led to more severe disease in our patient cohort? We observed that patients with more severe infection tended to be older individuals. This is in accordance with a study by Lee and colleagues from the UK Coronavirus Cancer Monitoring Project (UKCCMP), which showed that the all-cause-fatality rate in patients with cancer after Sars-CoV-2 infection is significantly associated with increasing age [5]. Similarly, Kuderer and colleagues concluded that elderly individuals with active cancer treatment have an increased risk of a serious complication. Additionally, they detected male sex as a prognostic variable associated with increased 30-day all-cause mortality [3]. We also noticed this effect in our cohort, as the majority of our cohort with a moderate or more severe infection were men. In fact, of the ten female patients, nine had a mild course. However, we evaluate this gender difference with caution as 53% of male patients had two or more comorbidities compared to 20% of female patients. Which leads us to our next consideration: the presence of comorbidities resulted in a more severe course of Covid-19. Despite having a small patient cohort, we saw that chronic diseases had a great impact on the course of Covid-19, especially type 2 diabetes mellitus and arterial hypertension [2, 8]. 75% of patients with a moderate, severe or critical course of Covid-19 had arterial hypertension, while only 30% of patients with a mild course had this comorbidity. In addition, the use of systemic corticosteroids and TNF alpha inhibitors prior to infection was documented more frequently in moderate to critical infection [4]. Especially in moderate infection, five out of six patients were on one of these immunomodulatory drugs.

Similar to the findings of Kuderer and colleagues, a higher number of comorbidities have been found to lead to more severe disease [3]. Our additional findings of advanced age and number of comorbidities correlated with an increased risk to severe infection is consistent with the general observations on this pandemic. For example, based on a meta-analysis or systematic review, the Center for Disease Control and Prevention (CDC) published a list of underlying diseases that are associated with a higher risk of severe Covid-19. These include cancer, pulmonary disease, diabetes mellitus, cardiac conditions, obesity, smoking and the use of corticosteroids or other immunosuppressive drugs [20].

A limitation of our study was certainly the small patient cohort. However, the experience of patients with metastatic melanoma and Covid-19 is of utmost importance for clinical practice. Since the onset of the pandemic, we tried to continue our established regimen for treating patients with melanoma to minimize any potential negative impact on the outcome of melanoma therapy. This was accompanied by some risk of more severe courses or even deaths from the virus. However, our data from the last two years of following 350 patients with metastatic melanoma show that patients mostly had a mild Covid-19 course despite immunotherapy or targeted therapy.

## MATERIALS AND METHODS

**Figure 2:**
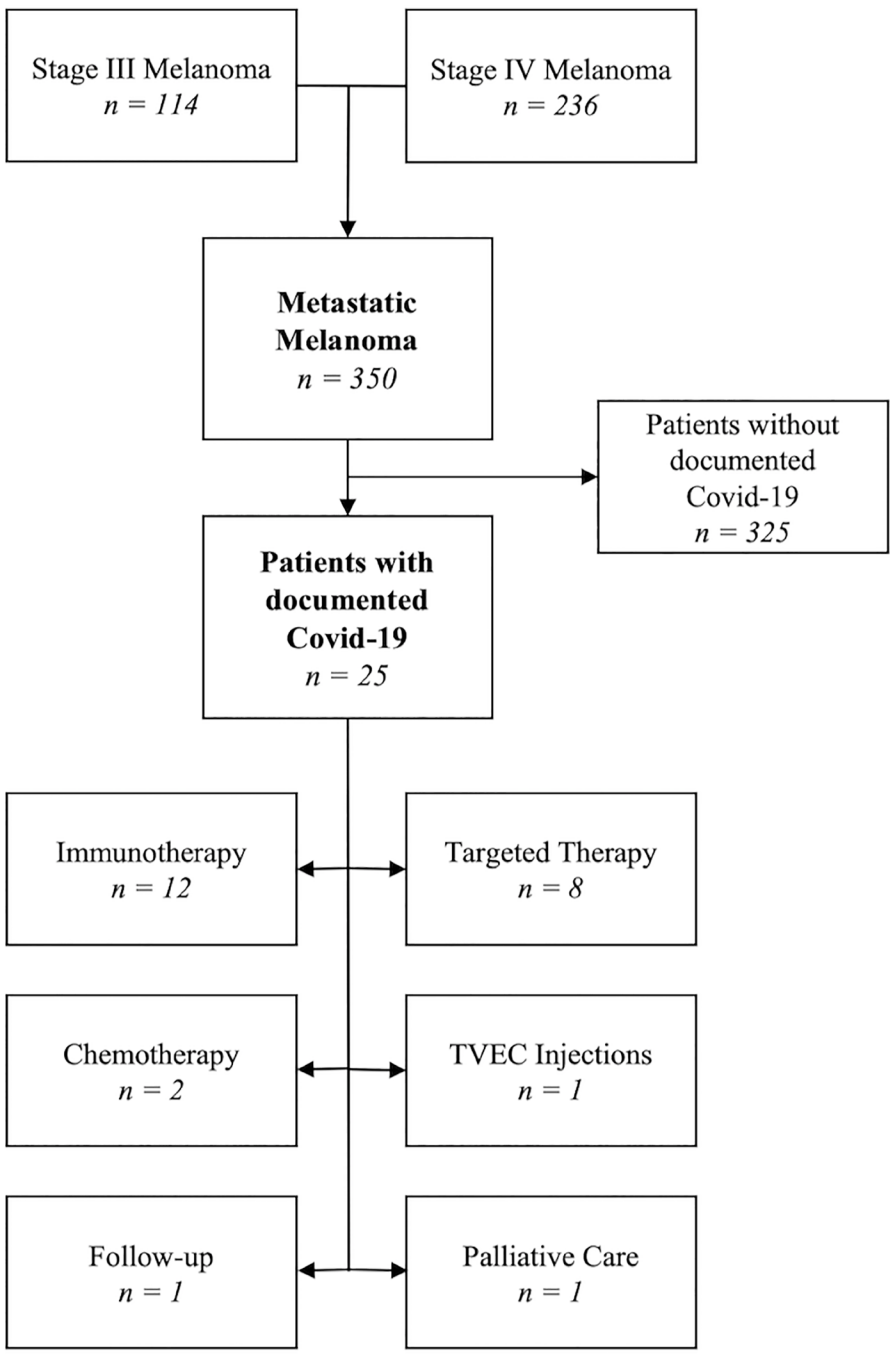
Flowchart for patients included in the analysis.

A retrospective cohort study was performed with the aim to analyze in more detail patients with metastatic melanoma and Covid-19. We included all patients undergoing treatment for metastatic melanoma (AJCC 8th Edition Stage 3 and 4) at the Department of Dermatology at the University Hospital Zürich from March 2020 until the end of February 2022, focusing on those with Covid-19 [21]. We assessed demographic data, cancer stage (TNM, AJCC 8th Edition stage), histological subtype, localization, Breslow thickness, BRAF mutation status and ECOG. We also recorded their treatment regime during Covid-19 (active treatment i.e. chemotherapy, targeted therapy, immunotherapy, follow-up, or palliative treatment) and the additional use of immunomodulatory drugs (i.e., steroids or tumor necrosis factor (TNF) alpha inhibitors). The following comorbidities were evaluated: arterial hypertension, other cardiovascular disease, type 2 diabetes mellitus, pulmonary disease (i.e., COPD, bronchial asthma), renal risk factors and obesity [2]. In the context of Covid-19, we recorded the date of infection, vaccination status, laboratory parameters before and after Covid-19 (i.e., leucocytes, lymphocytes, CRP and creatinine), severity of infection, need for hospitalization or treatment in the intermediate care unit (ICU) and if so, the duration. We classified the severity of the Sars-CoV-2 infection according to the Common Terminology Criteria for Adverse Events (CTCAE, v.5.0). We defined a mild infection as being asymptomatic or mildly symptomatic, such as acute upper respiratory tract infection, a moderate infection such as pneumonia with no obvious hypoxemia which needed minimal standard care, a severe infection such as pneumonia with hypoxemia which required hospitalization and significant medical care and a critical infection as life-threatening disease with for example an acute respiratory distress syndrome (ARDS) or other organ dysfunction which may lead to death [22, 23]. Due to the small patient cohort, we considered patients with a moderate to critical course as one group for statistical analyses. In addition, we analyzed whether there was a delay in melanoma treatment due to Covid-19 and the clinical outcome (i.e., alive, death from melanoma, death from Covid-19). The Cantonal Ethics Committee approved the collection of patient data, and all patients signed a general informed consent form. Figure 2 shows patients included in the study.

## CONCLUSIONS

With our analysis of patients treated for metastatic melanoma with Covid-19, we showed that despite the influence of cancer therapy, Sars-CoV-2 infection did not result in severe Covid-19 in most cases. The majority of patients had a mild course, even though the cancer had metastasized and they received systemic therapy. With this study, we hope to be able to contribute to patient management in a possible next wave of infection or even in the next pandemic. In our experience, it is important to continue immunotherapy or targeted therapy after an individual risk assessment, even in times with increased risk of infection with a new pathogen, while continuing to protect patients at risk through vaccination and extended hygiene measures.

## Abbreviations

ALM: acral lentiginous melanoma
ARDS: acute respiratory distress syndrome
AJCC: American Joint Committee on Cancer
BMI: body mass index
CDC: Center for Disease Control and Prevention
COPD: chronic obstructive pulmonary disease
CTCAE: Common Terminology Criteria for Adverse Events
Covid-19: Corona Virus Disease 2019
CCC19: Covid-19 and Cancer Consortium
CRP: C-reactive protein
CTLA-4: Cytotoxic T-Lymphocyte-Associated Protein 4
ECOG: Eastern Cooperative Oncology Group
ESMO: European Society for Medical Oncology
ICI: immune checkpoint inhibitors
irAEs: immune related adverse events
IT: immunotherapy
ICU: intermediate care unit
LMM: lentigo maligna melanoma
NM: nodular melanoma
NOS: not otherwise specified
PD-L1: Programmed Cell Death Ligand 1
PD-1: Programmed Cell Death Protein 1
Sars-CoV-2: severe acute respiratory syndrome coronavirus 2
SSM: superficial spreading melanoma
TVEC: talimogene laherparepvec
TT: targeted therapy
TNF: tumor necrosis factor
TNM: tumor nodes metastasis
UKCCMP: UK Coronavirus Cancer Monitoring Project
