# Addendum: Impact of Covid-19 on the management of patients with metastatic melanoma

**DOI:** 10.18632/oncotarget.28496

**Published:** 2023-09-25

**Authors:** Michèle Welti, Phil F. Cheng, Joanna Mangana, Mitchell P. Levesque, Reinhard Dummer, Laurence Imhof

**Affiliations:** ^1^Department of Dermatology, University Hospital Zurich (USZ), Zurich 8091, Switzerland; ^2^Faculty of Medicine, University of Zurich (UZH), Zurich 8032, Switzerland; ^3^Institute for Biomedical Engineering, ETH Zurich, Zurich 8092, Switzerland

**This article has an addendum:** The melanoma registry database of the Department of Dermatology has been partially supported by an unrestricted grant to the University of Zurich from Amgen, Novartis, BMS, MSD and Pierre Fabre. The study itself required no external funding.

Original article: Oncotarget. 2022; 13:1370–1379. 1370-1379. https://doi.org/10.18632/oncotarget.28333

